# Multimorbidity prediction using link prediction

**DOI:** 10.1038/s41598-021-95802-0

**Published:** 2021-08-12

**Authors:** Furqan Aziz, Victor Roth Cardoso, Laura Bravo-Merodio, Dominic Russ, Samantha C. Pendleton, John A. Williams, Animesh Acharjee, Georgios V. Gkoutos

**Affiliations:** 1 Institute of Cancer and Genomic Sciences, Centre for Computational Biology, University of Birmingham, Birmingham, B15 2TT UK; 2 Institute of Translational Medicine, University of Birmingham, Birmingham, B15 2TT UK; 3 NIHR Surgical Reconstruction and Microbiology Research Centre, University Hospital Birmingham, Birmingham, B15 2WB UK; 4 MRC Health Data Research UK (HDR UK), Birmingham, B15 2TT UK; 5 NIHR Experimental Cancer Medicine Centre, B15 2TT Birmingham, UK; 6 NIHR Biomedical Research Centre, University Hospital Birmingham, Birmingham, B15 2WB UK

**Keywords:** Bioinformatics, Computational models

## Abstract

Multimorbidity, frequently associated with aging, can be operationally defined as the presence of two or more chronic conditions. Predicting the likelihood of a patient with multimorbidity to develop a further particular disease in the future is one of the key challenges in multimorbidity research. In this paper we are using a network-based approach to analyze multimorbidity data and develop methods for predicting diseases that a patient is likely to develop. The multimorbidity data is represented using a temporal bipartite network whose nodes represent patients and diseases and a link between these nodes indicates that the patient has been diagnosed with the disease. Disease prediction then is reduced to a problem of predicting those missing links in the network that are likely to appear in the future. We develop a novel link prediction method for static bipartite network and validate the performance of the method on benchmark datasets. By using a probabilistic framework, we then report on the development of a method for predicting future links in the network, where links are labelled with a time-stamp. We apply the proposed method to three different multimorbidity datasets and report its performance measured by different performance metrics including AUC, Precision, Recall, and F-Score.

## Introduction

Multimorbidity is a prominent topic across healthcare research^1^. Despite the great gains in life expectancy over the last 150 years, a single disease approach is no longer providing proportionately progressive therapeutic improvements^2^. This is due to the fact that a majority of older adults have more than one long term condition—termed multimorbidity. It is characterized by a high degree of complexity. Conventional medical research, that often focuses on the study of diseases in isolation, is unlikely to accurately answer questions about patients suffering from many chronic conditions^2^. Multimorbidity is common in the elderly population, and associated with adverse outcomes, such as increased mortality, poor quality of life, and increased healthcare usage^1^. Almost 3 in 4 patients, aged 65 and above, suffer from multiple chronic conditions^3^. These patients form the primary users of health care services and are recognized as one of the major burdens on healthcare systems worldwide^4^. Although the term “multimorbidity” was first coined in 1976^5^, it has only gained popularity in the last decade^6^. Among other factors, one of the most important one is the rapid developments in artificial intelligence and machine learning based tools in recent years which has helped to answer many questions posed by multimorbidity research^7,8^. Among different available tools, network-based machine learning frameworks have received particular attention. The reason is that many diseases arise from common complex molecular interactions, sharing complex traits across them resulting in connections that can be systematically represented by networks^9^.

Network science is a rapidly growing field of interdisciplinary scientific research aiming at modelling various complex systems including biological systems using a complex network^10^. A biological system can be conveniently represented by means of a complex network, where nodes corresponds to biological entities such as patient, disease, phenotype and links represent complex interactions or association between those entities. For example, Halu et al.^9^ have build and analyzed a multiplex network of 779 human diseases, that consists of two different layers, namely a genotype-based layer and a phenotype-based layer. In this network, two diseases are linked if they share a common disease gene. Yu et al.^11^ have developed a Human Pathway-based Disease Network (HPDN) to explore the relationship between diseases and their intrinsic interactions. They observed that the similarity of two diseases has a strong correlation with the number of their shared functional pathways and the interactions between their related gene sets. In order to incorporate the temporal information and understand disease progression, LU et al.^12^ have designed a visual analytics system that is based on temporal disease network—a type of network with links that are assigned a set of time labels, indicating at which time steps the link is formed. By applying clustering algorithms to the underlying networks, they have studied the disease progression patterns in patients.

The different types of biological networks discussed above are generally categorized as unipartite networks, where there is only one type of node and any node can connect with any other node.Another type of network, termed bipartite network, has also been extensively researched in the domains of system biology and medicine^13–15^. A bipartite network is a network whose nodes can be grouped into two disjoint subsets, a source set and a target set. Links are allowed across the two sets but not within the same set. Such type of networks can naturally represent multimorbidity data. In this network representation, the two sets of nodes are the patient set and the disease set in which a link between a patient and a disease indicates the presence of the disease in the patient. Other examples of biological bipartite networks include enzyme reaction networks and gene-disease networks. Although a number of network analysis tools have been developed to analyze unipartite networks, the analysis of bipartite networks has received limited attention to date. The majority of network-based tools intended for unipartite network analysis may not be generalised to bipartite networks, since the structural properties of the latter are different from those of the former^16^. Consequently, many algorithms that exhibit good performance levels on unipartite networks may fail to characterise the structure of bipartite networks.

One of the most widely studied problems in complex network analysis is that of link prediction^17,18^. Link prediction approaches aim to estimate the likelihood of the existence of a link between disconnected nodes. Most of the commonly used link prediction methods, such as the common neighbour (CN)^19^, the preferential attachment (PA)^20^, and the resource allocation (RA)^21^ methods, are solely based on the local topological properties of a network. These methods, termed as local similarity indices, can be efficiently computed, and exhibit good performance in predicting missing links in many real-world networks. The other category is the global similarity indices that make use of the topological properties of the whole network^22^. Global methods generally outperform local ones, but they are computationally expensive rendering their application across large networks challengin g. A number of alternate approaches, that provide a trade-off between accuracy and computational time, have been proposed. Cannistraci et al.^23^ combined some popular local link prediction algorithms with a local community structure to define a new set of link prediction indices, called CAR-based indices, that proved useful in predicting missing links in brain connectomes. Lü et al.^24^ have proposed local path index that exploit of paths with wider horizon than common neighbour. Recently, we have proposed novel global and quasi-local indices of local link prediction methods and demonstrated their applications in real-world biological and social networks^25^.

Most link prediction methods were originally developed for unipartite networks. While some of the link prediction methods developed for unipartite graphs can be applied to bipartite graphs, most of the local link prediction algorithms cannot be directly adopted for bipartite networks due to the distinct topological structure of bipartite networks. To address this problem a number of methods have been developed to extend the prediction methods to bipartite networks. For example, based on the definitions of some local similarity indices for unipartite networks, Daminelli et al.^26^ developed variants of local similarity indices for bipartite networks including CN, JC, PA, AA, and RA. In addition, they also defined the notion of local community structure for bipartite networks as well as of CAR-based similarity indices for bipartite networks. The applications of the proposed method were demonstrated on various real-world datasets, including drug-target interaction networks. An alternate approach is formed by projection-based methods, where bipartite networks are typically first mapped to unipartite ones, which are then used to predict missing system interactions. For example, Kumar et al.^27^ have developed a framework for predicting missing links in a bipartite network using projection graphs. Their method for predicting links is based on two important concepts, namely potential energy and mutual information, which are computed between each pair of nodes in a projection graph. Recently, artificial neural network (ANN) are increasingly being employed for predicting missing links in bipartite networks. To this end, Shtar et al.^28^ have combined the classical similarity indices with ANN to predict missing links in drug-target interaction networks. In a related work, Guo et al.^29^ have developed graph neural networks to predict missing links in an bipartite network and explored its applications in biomedical networks including drug-target interaction networks.

In this paper we have developed a novel framework for predicting missing links in bipartite networks, and have explored its applications in biomedical datasets. Our novel contributions include the development of path-based similarity indices that can be used estimate the likelihood of existence of a missing link in a bipartite network. The approach adopted is based on our recent work^25^, where we have developed novel methods for predicting missing links in unipartite networks. Our method is efficient as it is based on paths of length 3, and we have validated its performance on four benchmark biomedical datasets. We further extend this method to temporal bipartite networks and develop similarity index that make use of conditional probabilities of the target set. Finally, we have developed a bipartite multimorbidity network. By applying this network across three different real-world multimorbidity datasets, we demonstrate that the proposed similarity index can exploit the topological structure of the resultant network to successfully predict future disease diagnosis.

## Methods and materials

We defined a local similarity measure to estimate the likelihood of existence of a link between two nodes in a bipartite network and extend it to a dynamic temporal bipartite network. A dynamic temporal bipartite network is a bipartite network that has the potential to change over time. In our case, we are considering a network whose links are time-stamped and the network evolves by adding more links with time. We then define the link prediction problem in complex network and discuss some commonly used link prediction methods.

### Overview of link prediction

A *network* $G = (V,E)$ is defined as a set of nodes, *V*, and a set of links, $E \subseteq V \times V$. A *bipartite network*, $G = {V_1, V_2, E}$ is a network whose nodes can be grouped into two disjoint subsets, $V_1$ and $V_2$, such that each link joins a node from one subset to another, but no two nodes in the same subset can be linked together. A unipartite network, on the other hand, is a network where every pair of nodes can possible interact with each other. An *adjacency matrix* provides a compact way of representing a network. For a bipartite graph $G = (V_1, V_2, E)$, its adjacency matrix is a matrix of size $\left| V_1\right| \times \left| V_2\right|$, whose (*u*, *v*) entry is 1 if node $x \in V_1$ is linked to node $y \in V_2$ and 0 otherwise. The degree of a node *v* is the number of connection that *v* forms with the nodes in the other set. We denote by $\left| \Gamma (v)\right|$, the degree of the node *v*, where $\Gamma (v)$ represents the set of all neighbours of *v*. Clearly, if $v \in V_1$, then $\Gamma (v) \subseteq V_2$ and, equivalently, if $v \in V_2$, then $\Gamma (v) \subseteq V_1$. We further denote by $\widehat{\Gamma }(v)$, the union set of all nodes, that are connected to the neighbours of the node *v*. Mathematically, this set can be defined as $\widehat{\Gamma }(v) = \bigcup _{z \in \Gamma (v)} \Gamma (z)$. A walk of length *k* in a bipartite network is the sequence of nodes $v_0, v_1, v_2,\dots, v_k$ where $v_i \in V_1 \cup V_2, \forall i=0,1,2,\dots,k$ and $(v_{i-1},v_{i})\in E, \forall i = 1,2,\dots,k$.

The definition of common neighbour index in a unipartite network is based on the notion of the triadic closure principle (TCP). This principle states that, among three nodes, *u*, *v*, and *w*, if *u* is connected with *v* and *v* is connected with *w*, then *u* and *w* have an increased probability of being connected. In other words, the nodes *u* and *w* are likely to be connected, if they share a common neighbour. Based on TCP, the CN index estimates the likelihood of existence of a link between two disconnected nodes by computing the count of their common neighbours. Mathematically, this index can be expressed as $Z_{uv}^{CN} = \left| \{\Gamma (u) \cap \Gamma (v)\}\right|$. The CN index, originally defined for unipartite networks, cannot be directly applied to bipartite networks to predict missing links. This is due to the fact that, in a bipartite network, a link is always formed between nodes of different subset and such nodes do not share any common neighbours. Consequently, the CN index will always result in assigning a zero score to every pair of disconnected nodes. Therefore, there is a need to revise the local similarity indices for bipartite networks.

One of the earliest attempts to develop local link prediction algorithms for bipartite networks was proposed by Huang et al.^30^. They have revised the definition of various local link prediction indices for bipartite networks and have explored their applications in collaborative filtering. These indices were also proved useful in predicting links in drug-target interaction networks^31^. According to^30^, two nodes $u \in V_1$ and $v \in V_2$ are likely to connect if the neighbours of node *u* and node *v* share many common neighbours. Mathematically, CN can be defined as follows:

1 $$\begin{aligned} S_{uv}^{CN1} = |\widehat{\Gamma }(u) \cap \Gamma (v)|, u \in V_1, v \in V_2. \end{aligned}$$

From the above equation, it is obvious that CN index is defined using paths of length 3 between two nodes *u* and *v* to estimate the likelihood of existence of a link between them. However, Eq. 1 only takes into account the frequency of those nodes that belong to set $V_1$.

The authors have also revised the bipartite versions of other local similarity indices including JC, AA and PA. We note that, while the CN index defined in Eq. 1 counts the number of common neighbours in $V_1$, an alternate way to define the CN index is by using common neighbours in $V_2$. This index can be defined as follows:

2 $$\begin{aligned} S_{uv}^{CN2} = |\Gamma (x) \cap \widehat{\Gamma }(y)|, x \in V_1, y \in V_2. \end{aligned}$$

**Figure 1:**
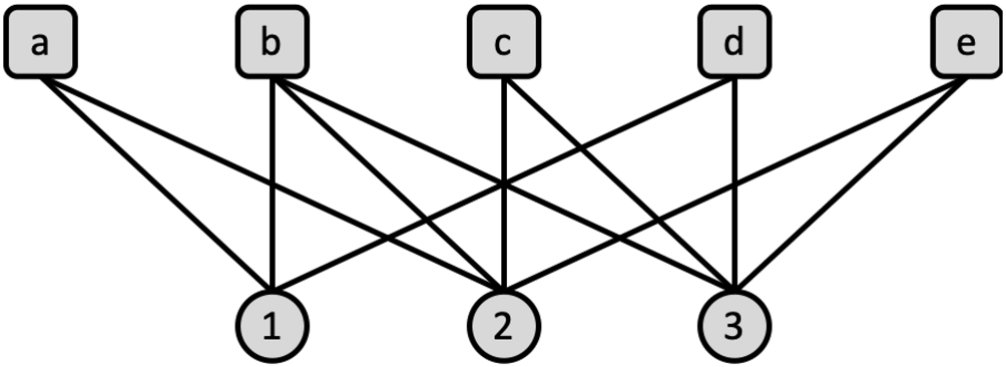
An example of a bipartite graph with 8 nodes and 11 edges.

Since the two similarity indices defined in Eqs. 1 and 2 are based on the count of nodes that belong to different sets, therefore they may produce different ranking scores of missing links. This is demonstrated in the following example. Consider the graph $G = (V_1,V_2,E)$, with 8 nodes and 11 edges shown in Fig. 1, where $V_1=\{a,b,c,d,e\}$ and $V_2 = \{1,2,3\}$.

Suppose we want to find the likelihood of existence of a link between the two nodes $e \in V_1$ and $1 \in V_2$. Then the similarity score would be according to Eq. 1$S_{uv}^{CN} = |\widehat{\Gamma }(e) \cap \Gamma (1)| = 3$, while according to Eq. 2, $S_{uv}^{CN1} = |\Gamma (e) \cap \widehat{\Gamma }(1)| = 2$. Therefore, the similarity score computed, using two different methods, may result in different rankings of predicted links. This is obvious, as the similarity score $S_{uv}^{CN1}$ is computed using all the common neighbours that belong to set $V_1$, while $S_{uv}^{CN2}$ is computed using all the common neighbour that belong to set $V_2$. Therefore, there is a need to define similarity indices for bipartite networks that are independent of the sizes of $V_1$ and $V_2$. To overcome this problem, Daminelli et al.^26^ have defined common neighbour index for a bipartite graph that takes into account the information of all nodes lying on paths of length three between the two nodes. The bipartite version of the common neighbour index is defined as follows^26^:

3 $$\begin{aligned} S_{uv}^{CN} = \left| \{\widehat{\Gamma }(x) \cap \Gamma (y)\} \cup \{\Gamma (x) \cap \widehat{\Gamma }(y)\}\right| , x \in V_1, y \in V_2. \end{aligned}$$

The index defined in Eq. 3 predicts the likelihood of (*x*, *y*)-interaction by counting the number of neighbours touched by quadrangles that pass through the nodes *x* and *y*. Note that, since, the two sets $S_{uv}^{CN1}$ and $S_{uv}^{CN2}$ are disjoint sets, it can be shown that $S_{uv}^{CN} = S_{uv}^{CN1} + S_{uv}^{CN2}$. Therefore, according to Eq. 3, for the bipartite graph of Fig. 1, $S_{uv}^{CN} = 3+2 = 5$. The definition of a common neighbour index allows to revise other related variations of common neighbour index including JC, AA, RA, and PA. For instance, the AA index for a bipartite network is defined as follows^26^:

4 $$\begin{aligned} S_{uv}^{AA} = \sum _{z\in \{\{\widehat{\Gamma }(x) \cap \Gamma (y)\} \cup \{\Gamma (x) \cap \widehat{\Gamma }(y)\}\}} \frac{1}{\log _2\left| \Gamma (z)\right| } \end{aligned}$$

One of the limitations of the revised local similarity indices for bipartite graph is that they do not take into account the connectivity structure between the neighbour nodes of the two query nodes. In order to overcome this limitation with the classical local similarity indices, the authors have also defined local-community-paradigm (LCP) for bipartite networks^26^. The idea of LCP was first introduced by Cannistraci et al.^23^ for unipartite networks. According to this theory, the similarity score between two disconnected nodes is not only computed using the count of the common neighbours, but also takes into account the organisation of the links between all the common neighbours. The concept of Local community links (LCL) between two disconnected nodes *x* and *y* is defined as follows:

5 $$\begin{aligned} S_{uv}^{LCL} = \left| \{(u,v): (u,v) \in E, u \in \Gamma (y), v \in \Gamma (x)\}\right| \end{aligned}$$

Informally, the value $S_{uv}^{LCL}$ represents the number of links present in the cohort formed by the neighbours of the nodes *x* and *y*. The definition of LCL has allowed the authors to revise the classical local similarity indices including CN, AA, JC, RA, and PA. These new revised similarity indices are termed CAR-base similarity indices. Daminelli et al.^26^ have extended the concept of CAR-based similarity indices to bipartite networks. They have defined the bipartite version of the CAR-based version of local similarity indices including CN, AA, JC, RA, and PA. The proposed indices were applied to networks originating from different domains including technological, social and biological systems. The local similarity indices for unipartite graph and the corresponding similarity indices for bipartite graphs, defined in^26^, are reported in Table S1 in the supplementary material. .

### Path-based similarity index

In an attempt to overcome the limitations of existing local similarity indices for bipartite network, we define a local similarity index for a bipartite network that is based on unique paths of length 3. The advantage of the proposed framework is that, not only, it allows us to revise the local link prediction indices to bipartite network, but it also caters the definition of a novel path-based index for predicting future links in a dynamic temporal bipartite network. The advantage of computing local indices using path-based information is that it can capture the local topology of a network around a node more accurately. For example, Fig. 1 presents 4 unique paths of length 3, i.e., $\{e, 2, a, 1\}$, $\{e, 2, b, 1\}$, $\{e, 3, b, 1\}$, and $\{e, 3, d, 1\}$, between the nodes *e* and 1, while their common neighbour similarity score is $S_{uv}^{CN} = 5$. By adding the edge (*a*, 3) in this graph, the number of unique paths of length 3 between nodes *e* and 1 increase by 1. However, their common neighbour score remains the same. CAR-based common index is an attempt to incorporate the path information, as it can be shown that the measure LCL is equal to the number of unique paths of length 3 between the two disconnected nodes. The definition of LCL has allowed the authors to define the CAR-base version of other similarity indices.

In our recent work^25^, we have developed methods for defining path-based extensions of some of the local similarity indices for unipartite graphs. The advantage of our approach is that it not only considers paths with wider horizons, but also takes into account the contribution of each node on those paths. We have empirically demonstrated that the proposed similarity indices can not only improve the predication accuracy of classical link prediction methods but can also outperform other state-of-the-art link prediction similarity indices including CAR-based indices. We have further demonstrated that, among the path-based extensions of local similarity indices, the path based-extension of the RA index generally outperforms all the alternate methods. In this paper, we use a similar approach to define the path-based resource allocation (PRA) index for bipartite graphs. The proposed approach is based on the unique paths of length 3 between two nodes $u \in V_1$ and $v \in V_2$ and is defined as follows:

6 $$\begin{aligned} S_{uv}^{PRA} = \sum _{(u,v): (u,v) \in E, u \in \Gamma (x), v \in \Gamma (y)} \frac{1}{\left| \Gamma (u) \right| \times \left| \Gamma (v) \right| }. \end{aligned}$$

Next, we revise this index for temporal bipartite networks and explore its applications in predicting diseases in patients with multimorbidity (defined as patients suffering from two or more diseases). The objective here is to estimate the likelihood of a patient developing a new disease in the future, given that they already suffer from a set of known diseases. This problem can be conveniently solved using bipartite network representation. We assume that there are two types of nodes in the network, the patient set ($\mathcal{P}$) and the disease ($\mathcal{D}$). All the links are time stamped and are only allowed between patients and diseases. Predicting new diseases in patient would be equivalent to prediction of new links in the network. Suppose, we want to predict a link between a node $p \in \mathcal{P}$ and $d \in \mathcal{D}$. Our approach of predicting new links in the network is based on paths of length three and the following assumption: Let $\{p,d',p',d\}$ be a path of length 3 in the network, where $p' \in \mathcal{P}, p'\ne p$ and $d' \in \mathcal{D}, d' \ne d$. Let $\Pr (p=d | p=d')$ be the probability that *p* develops *d* in future given that they have already been diagnosed with disease $d'$. Then a path with a higher value of $\Pr (p=d | p=d')$ is likely to contribute more to the similarity score than a path with a lower value of of $\Pr (p=d | p=d')$. Based on this hypothesis, we revise Eq. 6 for a temporal bipartite network as follows:

7 $$\begin{aligned} S_{pd}^{TPRA} = \sum _{(d',p'): (d',p') \in E, d' \in \Gamma (p), p' \in \Gamma (d)} \frac{\Pr (p'=d | p'=d')}{\left| \Gamma (p') \right| \times \left| \Gamma (d') \right| }. \end{aligned}$$

In other words, this index can be considered as a weighted version of the path-based similarity index, where paths are weighted by the inverse of the degrees of internal nodes on the paths and conditional probabilities of the nodes. We demonstrate the computation of this index with an example. Consider again, the bipartite graph shown in Fig. 1. Suppose the sets $V_1=\{a,b,c,d\}$ and $V_2=\{1,2,3\}$ represent the patient and the disease sets respectively, and we want to estimate how likely is patient $e \in V_1$ to develop disease $1 \ in V_2$ in future. Then according to Eq. 7, the similarity score, $S_{e1}^{TPRA}$, can be computed as:

8 $$\begin{aligned} S_{e1}^{TPRA} = \sum _{(p',d')\in \{(a,2),(b,2),(b,3),(d,3)\}} \frac{ Pr(p'=1|p'=d')}{\left| \Gamma (d) \right| \times \left| \Gamma (3) \right| }, \end{aligned}$$

which can be simplified to give us:

9 $$\begin{aligned} S_{e1}^{TPRA} = \frac{\Pr (a=1 | a=2)}{\left| \Gamma (a) \right| \times \left| \Gamma (2) \right| } + \frac{\Pr (b=1 | b=2)}{\left| \Gamma (b) \right| \times \left| \Gamma (2) \right| } + \frac{\Pr (b=1 | b=3)}{\left| \Gamma (b) \right| \times \left| \Gamma (3) \right| } + \frac{\Pr (d=1 | d=3)}{\left| \Gamma (d) \right| \times \left| \Gamma (3) \right| }. \end{aligned}$$

In the above equation, the similarity score is computed using four different terms, where each term corresponds to a unique path of length 3 in the network. It is worth noting that the time complexity of the proposed indices is similar to that of the time complexities of alternate method defined earlier in this section. This is because all the methods are based on the information about the immediate neighbours of the two query nodes. This makes it practical to apply the proposed similarity indices on larger bipartite networks. To understand how the proposed similarity index is computed, a numerical example, demonstrating the computation of the proposed similarity index, is provided in the supplementary material.

### Datasets

To assess the performance of the path-based local similarity index proposed in Eq. 6, we use drug-target interaction (DTI) networks. A DTI network is a bipartite network with two types of nodes, the drug molecules and the target proteins. Since in vitro methods (biochemical experiments) for finding drug–target interaction are extremely costly and time-consuming^32^, in silico methods have gained substantial popularity for efficiently predicting potential interactions^33^. Here, we are using four classes of drug–target interaction networks^34^, involving nuclear receptors (NR)^35^, G-protein-coupled receptors (GPCRs)^36^, ion channels (IC)^37^, and enzymes (EZ)^38^ . These networks are publicly available and are considered benchmark datasets for link prediction methods for bipartite networks^33^. The statistical properties of these networks are reported in Table 1.

**Table 1:** Topological properties of the networks used. Here, |*V*| and |*E*| are the number of nodes and links respectively, $|V_1|$ and $|V_2|$ are the number of nodes in the left and the right side of the bipartite graph respectively, and $\left<k^{V_1}\right>$ and $\left<k^{V_2}\right>$ are the average degrees of the nodes in the sets $V_1$ and $V_2$ respectively.

Dataset	$\left| V\right|$	$\left| V_1\right|$	$\left| V_2\right|$	$\left| E\right|$	$\left<k^{V_1}\right>$	$\left<k^{V_2}\right>$
Nuclear receptors	80	26	54	90	3.46	1.67
G Protein-coupled receptors	318	95	223	635	6.68	2.85
Ion Channels	414	204	210	1476	7.24	7.03
Enzymes	1109	664	445	1109	1.67	2.49
Multimorbidity dataset 1 (MM1)	33,805	33,736	69	175,921	5.21	2549.60
Multimorbidity dataset 2 (MM2)	10,696	10,634	62	59,696	5.61	962.84
Multimorbidity dataset 3 (MM3)	33,986	33,958	28	171,330	5.04	6118.90

To evaluate the performance of the path-based local similarity index for temporal bipartite graphs, proposed in Eq. 7, we have constructed a temporal bipartite network from three different multimorbidity datasets that can be obtained from the UK Biobank project 31224—*Explanatory epidemiological models from genotype to phenotype*. In each of these multimorbidity datasets, every patient has a known set of diseases with defined diagnosis date. Our bipartite representation of the multimorbidity dataset encapsulates two types of nodes, the patient and the disease nodes. A link established between a patient *p* and a disease *d*, if *d* is reported in *p*. The edges are labelled with the diagnosis date. There are 69 diseases in the first dataset, 62 in the second dataset, and 28 in the third dataset. So as to improve the performance and the link-prediction accuracy, we only selected those patients who were diagnosed with at least four different conditions. The statistical properties of these three networks are reported in the last two rows of Table 1.

### Evaluation metric

We use two different standard evaluation metrics, the area under the receiver operating characteristic curve (AUC)^39^ and precision^40^, to estimate the accuracy of a link prediction algorithm. Let us consider a simple bipartite network $G = (V,E)$. Here we refer to the set of links, *E*, present in the network, as the set of observed links. Let $E'$ represent the set of nonexistent links in the network. In other words, $E' = \{(u,v): u \in V_1, v \in V_2, (u,v) \notin E\}$. We note that, if *U* represents the set of all possible $|V_1|\times |V_2|$ links that *G* can have, then $E' = U \setminus E$. In order to evaluate the prediction algorithm’s performance, the set of observed links, *E*, is divided into two disjoint sets, a training set $E^T$ and a probe set $E^P$. For the static bipartite networks, this division is randomly performed and all the links have same probability to picked as a training or a test link. For the temporal networks, first a set of patients is randomly selected. Then for each patient *p*, the link (*p*, *d*) with highest time value is added to the probe set $E^P$, while the remaining links are added to the set $E^T$. This is done in order to predict the performance of a link prediction algorithm for predicting future links. The links of all the remaining patients, that were not chosen in the random selection, are also added to the set $E^T$. Since $E^T$ and $E^P$ are disjoint, the two sets form a partition of the set *E*, i.e., $E = E^T \cup E^P$, and $E^T \cap E^P = \phi$. The information in $E^T$ is used to predict missing links while the information in $E^P$ is used to evaluate the performance of the prediction algorithm. This process is demonstrated in Figure S1 (supplementary material). Note that the split of observed links into a training set and a probe set is performed in the same way as it is performed for unipartite graphs in the literature^39,41^. To obtain an unbiased estimate of performances, all the experiments were repeated 100 times. In each run of the experiment, an independent random sampling of the observed links into test and probe set was performed. For each method and each dataset, we have reported the average accuracies along with the standard deviation values of all the runs. To estimate the performance, two metrics are used AUC and precision are used.

The metric AUC is the probability that a randomly chosen link in $E^P$ gets higher score than a randomly chosen link in $E'$. Here, among *n* independent comparisons, $n'$ is the number of times a missing link has higher score than a non-existent link, and $n''$ is the number of times a missing link and a nonexistent link having the same score and the *AUC* is defined as^39^:

10 $$\begin{aligned} AUC = \frac{n'+0.5n''}{n}. \end{aligned}$$

We note that the AUC value should be roughly 0.5, if all the link scores are randomly generated according to an independent identical distribution. Therefore, an AUC value greater than 0.5 indicates how well the prediction algorithm performs when compared to pure chance.

Precision is defined as the ratio of relevant items selected to the number of items selected. We also report the value of Recall and F-Score. Recall is defined as the ratio of correctly predicted items to all the positive items, while the metric F-Score is defined as follows:

11 $$\begin{aligned} \text {F-Score} = \frac{2\times p \times r}{p + r}, \end{aligned}$$

where *p* and *r* represent the precision and recall values respectively.

## Results and discussion

We applied the path-based resource allocation similarity index, proposed in Eq. 6, to four PPI networks. To evaluate the performance of the proposed method, we compared its performance with the performance of five classical local similarity indices and the six CAR-based similarity indices discussed in Section 2.1. To estimate the prediction accuracy of all the prediction indices, we split the observed links into a training set $E^T$ and a probe set $E^P$, such that 90% of the links belong to the training set while the remaining 10% links lie within the probe set. The performance of all the similarity indices were estimated using the same training and probe sets. The average AUC values along with the standard deviation values of all the 100 runs are reported in Table 2.

**Table 2:** AUC values of the proposed and alternate methods for the four DTI datasets. Each experiment was executed 100 times and the average values along with the standard deviations are reported.

Method	NR	IC	GPCR	EZ
CN	0.6967 ± 0.0707	0.9060 ± 0.0135	0.8405 ± 0.0246	0.8842 ± 0.0115
JC	0.6830 ± 0.0658	0.8570 ± 0.0114	0.8348 ± 0.0242	0.8798 ± 0.0112
AA	0.6961 ± 0.0704	0.9132 ± 0.0135	0.8480 ± 0.0255	0.8853 ± 0.0115
RA	0.6961 ± 0.0704	0.9150 ± 0.0134	0.8499 ± 0.0260	0.8857 ± 0.0115
PA	0.4971 ± 0.1092	0.8258 ± 0.0171	0.7186 ± 0.0412	0.7728 ± 0.0184
LCL	0.6970 ± 0.0708	0.9131 ± 0.0130	0.8396 ± 0.0245	0.8850 ± 0.0116
CAR	0.6969 ± 0.0708	0.9117 ± 0.0133	0.8402 ± 0.0245	0.8850 ± 0.0115
CJC	0.6942 ± 0.0696	0.9115 ± 0.0126	0.8428 ± 0.0243	0.8850 ± 0.0115
CAA	0.6973 ± 0.0707	0.9193 ± 0.0132	0.8455 ± 0.0250	0.8861 ± 0.0115
CRA	0.6977 ± 0.0712	0.9233 ± 0.0132	0.8487 ± 0.0255	0.8867 ± 0.0115
CPA	0.6975 ± 0.0711	0.9122 ± 0.0128	0.8392 ± 0.0247	0.8850 ± 0.0115
PRA	**0.6984 ± 0.0713**	**0.9280 ± 0.0135**	**0.8521 ± 0.0260**	**0.8872 ± 0.0115**

Bold values indicate best performance.

The average AUC values of the proposed method and alternate local link prediction methods, reported in Table 2, suggest that the incorporation of the path-based information results in better predictions across all datasets. We note that the AUC values for the NR dataset are very low when compared to AUC values obtained for other datasets. In addition, the variations values for the NR dataset are high. This is due to the small size of the NR network. We next evaluate the performance of the proposed method and those of the alternative methods by computing the precision with the same split between the training and probe sets. The resulting precision values along with Recall and F-Score values are reported in Tables S2, S3, S4, S5 in supplementary material. These results demonstrate the usefulness of the proposed similarity index in predicting missing links in bipartite networks.

**Figure 2:**
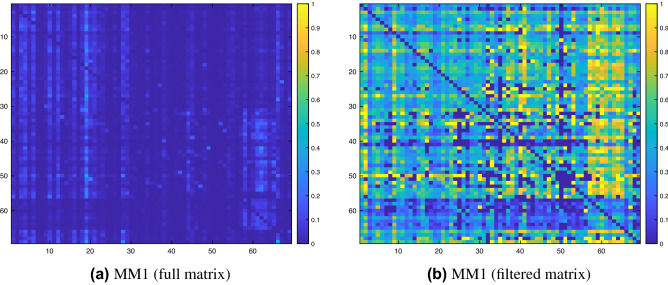
Each matrix entry, *M*(*i*, *j*), represents the conditional probability $Pr(patient=j | patient=i)$, ie., the probability that a patient is diagnosed with a disease *j*, given that they already suffer from disease *i*. The left panel plots data for all patients, while the right shows only those patients are considered who were diagnosed with both the diseases *i* and *j*. In other words, given that a patient has been diagnosed with both diseases *i* and *j*, how likely is that the patient was diagnosed with disease *i* before the patient was was diagnosed with disease *j*.

For our next set of experiments, we evaluated the performance of path-based index to predict missing links in temporal bipartite networks. We applied the proposed similarity index (Eq. 7) as well as the alternative methods over the two patient-disease networks discussed earlier. Before applying the link prediction method, we first computed and plotted the conditional probability matrix ’*M*’, whose $(i,j)^{th}$ entry represents the probability, $Pr(p=j | p=i)$, that a patient develops a disease *j* in future given a disease *i* diagnosis. The probability matrix for the *MM*1 dataset is plotted in the left column of Fig. 2. For the other two datasets, the similarity matrices are shown in Figure S1, supplementary material. We note that the conditional probabilities are not very high in most cases, which may be due to incomplete data. For instance, it is possible that a patient who has been diagnosed for a disease *j* is likely to develop a disease *i* in future but has not yet been tested for the disease *i*. To investigate it further, we have only considered those patients who are diagnosed with both the disease *i* and disease *j* and have computed the conditional probabilities. The probability matrix for the first filtered dataset is plotted in the right column of Fig. 2, while the probability matrices for the remaining two filtered datasets are shown in the right column of Figure S1, supplementary material. The high conditional probabilities values shown in this table suggest that, for some pair of diseases (*i*, *j*), it is highly likely that a patient is going to develop a disease *j* given that they are already diagnosed with disease *i*.

We demonstrate the effectiveness of the proposed similarity index (Eq. 7) for predicting missing links in a temporal bipartite network. To evaluate the performance, we applied the proposed and the alternate methods to the patient-disease networks. We first estimate the prediction accuracy of a link prediction method using AUC. As with the previous experiments, we divided the set of the observed links into two sets, the training set $E^T$ and the probe set $E^P$, such that 90% of the links are in $E^T$ while the remaining 10% links are in $E^P$. The conditional probabilities are computed from the training data. The estimated accuracies of the proposed and alternative indices for all the three datasets, measured by AUC (Eq. 10), are reported in Table 3. As with previous experiments, the AUC values reported here are the average of 100 independent runs. We have observed the AUC values of all the different methods for all the three networks become stable after around 20 iterations. For MM2, the smallest network with 10696 links, we have demonstrated this in Figure S4, supplementary material.

**Table 3:** AUC values of the proposed and alternate methods for the three multimorbidity datasets. Each experiment was executed 100 times and the average values along with the standard deviations are reported.

Method	MM1		MM2		MM3	
	AUC	AUROC	AUC	AUROC	AUC	AUROC
CN	0.8215 ± 0.0012	0.8329 ± 0.0011	0.8101 ± 0.0018	0.8254 ± 0.0017	0.7584 ± 0.0012	0.7978 ± 0.0010
LCL	0.8184 ± 0.0012	0.8302 ± 0.0011	0.8018 ± 0.0019	0.8179 ± 0.0017	0.7480 ± 0.0011	0.7892 ± 0.0009
RA	0.8201 ± 0.0012	0.8317 ± 0.0011	0.8071 ± 0.0019	0.8227 ± 0.0017	0.7556 ± 0.0012	0.7955 ± 0.0010
AA	0.8207 ± 0.0012	0.8322 ± 0.0011	0.8085 ± 0.0019	0.8239 ± 0.0017	0.7565 ± 0.0012	0.7963 ± 0.0010
CRA	0.8181 ± 0.0012	0.8299 ± 0.0011	0.8012 ± 0.0019	0.8173 ± 0.0017	0.7479 ± 0.0012	0.7892 ± 0.0010
CAA	0.8191 ± 0.0012	0.8309 ± 0.0011	0.8022 ± 0.0019	0.8182 ± 0.0017	0.7482 ± 0.0012	0.7894 ± 0.0010
CPA	0.8205 ± 0.0012	0.8322 ± 0.0011	0.8067 ± 0.0018	0.8224 ± 0.0017	0.7547 ± 0.0012	0.7949 ± 0.0010
JC	0.6340 ± 0.0013	0.6588 ± 0.0012	0.5392 ± 0.0023	0.5768 ± 0.0021	0.5481 ± 0.0018	0.6216 ± 0.0015
PA	0.7975 ± 0.0012	0.8109 ± 0.0011	0.7896 ± 0.0018	0.8065 ± 0.0017	0.7296 ± 0.0010	0.7743 ± 0.0009
CAR	0.8205 ± 0.0012	0.8321 ± 0.0011	0.8067 ± 0.0019	0.8223 ± 0.0017	0.7547 ± 0.0012	0.7949 ± 0.0010
CJC	0.7990 ± 0.0013	0.8123 ± 0.0012	0.7812 ± 0.0020	0.7989 ± 0.0018	0.7330 ± 0.0011	0.7768 ± 0.0009
PRA	0.8483 ± 0.0010	0.8579 ± 0.0010	0.8153 ± 0.0016	0.8303 ± 0.0015	0.7508 ± 0.0010	0.7916 ± 0.0009
PROP	**0.8604 ± 0.0010**	**0.8691 ± 0.0009**	**0.8420 ± 0.0016**	**0.8546 ± 0.0015**	**0.7747 ± 0.0011**	**0.8115 ± 0.0009**

Bold values indicate best performance.

**Figure 3:**
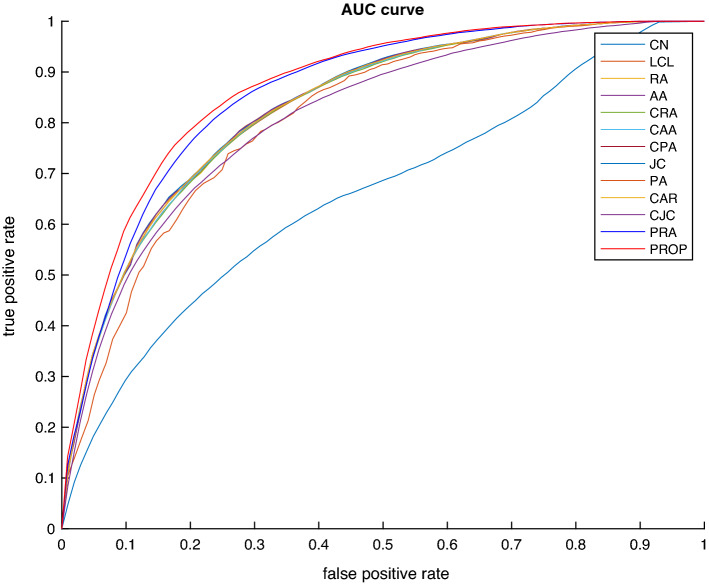
Comparison of the receiver operating characteristic curves of different methods when applied to the MM1 dataset.

The classification AUC of the proposed and of the alternative methods, reported in Table 3, suggest that the proposed approach is very effective in predicting missing links in temporal bipartite networks. To further investigate the performance of the proposed method, we have also plotted the receiver operating characteristic curve for the proposed and the alternate methods for the first dataset in Fig. 3. For the remaining two dataset, the ROC curve is shown in Figure S2, supplementary material. For each method, we have estimated area under the receiver operating characteristic (AUROC), and have reported the results in Table 3. Finally, we have estimated the performance of the proposed and alternative methods using precision, recall, and F-Score. The values for the datasets MM1, MM2, and MM3 are reported in Tables S6, S7, and S8 respectively in the supplementary material. Although, the precision values are low for all the three datasets, the proposed similarity index still achieves the highest precision in all cases. These results suggest that the proposed method is highly effective in predicting missing links in a dynamic temporal network.

**Figure 4:**
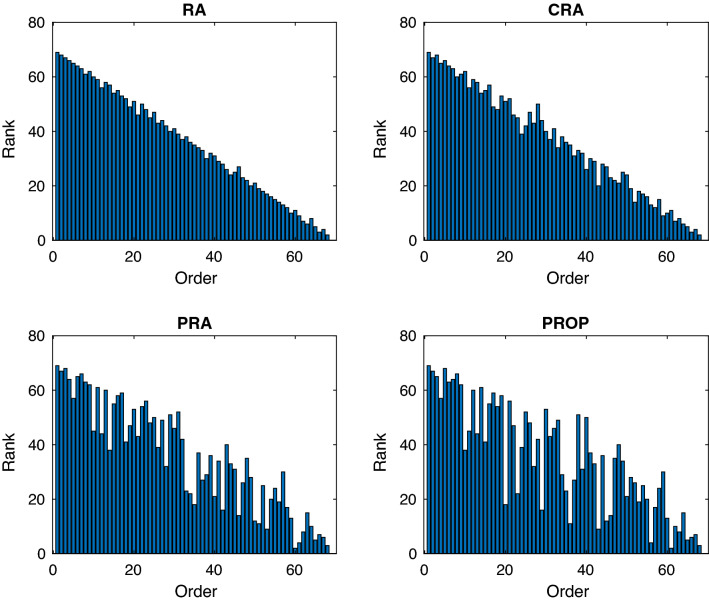
Prediction of new diseases for the multimorbidity dataset MM1. The x-axis shows the time when a new disease is predicted for the first time, while the y-axis shows the rank of the predicted disease when sorted using degree of disease in the network.

Many classical link prediction indices for bipartite networks suffer from the tendency of assigning high scores to those interactions whose source or target node has a higher degree in the network. These methods may fail to predict links whose end nodes have low degree in the network. One of the advantages of path-based link prediction method is that their ranking of missing links is not biased towards the degree of the nodes. To understand this, we present the order in which a disease is correctly predicted for the first time by the four link prediction methods, namely RA, CRA, PRA and PROP, in Fig. 4. Here, the x-axis shows the order in which the disease is predicted correctly for the first time, while the y-axis shows the ranking of the disease when sorted in ascending order by its degree in the network. The plots are shown for the dataset MM1, while similar plots for datasets MM2 and MM3 are respectively shown in Figures S3, and Figures S4, in supplementary material. The plots for the remaining similarity indices are also shown in the supplementary material in Figures S5, Figure S6, and Figures S7 for the datasets MM1, MM2 and MM3 respectively. These plots show that the classical, as well as the CAR-based similarity indices (except for JC indices), are more sensitive to the degree of the node, when missing links are sorted according to their similarity scores. The JC index is independent of the degree of the node, but it suffers from low performance. On the other hand, the PRA and the PROP similarity indices not only give better performances but are also less sensitive to the degree of the nodes.

To further investigate these results, we look at the number of unique diseases correctly predicted by each method in the three multimorbidity datasets. For each link prediction algorithm method, we have sorted the predicted links by their ranking scores and selected the top 50% of the links correctly predicted by each method. In Table 4, we report the count of unique diseases correctly predicted by each method, for each of the 10 independent runs of the algorithm. A scalar value *x* in the table represents the case when the count of unique diseases remains the same in different independent runs, while the pair $x-y$ represents the range (minimum and the maximum values) of the count obtained in different runs.

**Table 4:** Rows 1–3 (not counting the header row) of the top partition refers to counts of unique diseases. The bottom three rows give the order of the lowest disease predicted by each method, when diseases are sorted in descending order by their node degree. The bold shows that the similarity indices suffer from low prediction accuracies.

Method	CN	LCL	RA	AA	CRA	CAA	CPA	JC	PA	CAR	CJC	PRA	PROP
MM1	13–14	17–20	14–15	13–14	19–21	18–21	16–16	**66–68**	**26–29**	16–16	**23–27**	32–34	26–28
MM2	16	22–23	16–17	16	22–25	22–23	20–21	**58–60**	**23–25**	20–21	**27–29**	26–27	19–20
MM3	10	14–16	9	9–10	14–16	14–16	12	**27**	**17–18**	12	**15–16**	16–18	14–15
MM1	14–14	22–23	14–15	14–14	22–23	22–23	16–16	**67–68**	**28–31**	16–16	**25–31**	47–48	51–52
MM2	16	27	16–17	16	22–27	27	20–22	**60–61**	**23–27**	20–22	**30–36**	35–36	23–25
MM3	10	15–16	9	9–10	15–16	15–16	12	**27**	**18**	12	**16**	16–18	15

The results reported in Table 4 show that the JC index is able to predict highest number of unique disease in a network. However, as reported earlier, the JC index suffers from a very low prediction accuracy, when measured by both the AUC and precision, depicting its tendency to predict many false-positive links in a network. The other two indices with low prediction accuracies are PA and CJC. These three indices are highlighted in bold in the table. From the remaining ten indices, the PRA index is the most successful in predicting the highest number of unique diseases in all the three datasets. In the bottom three rows of the table, we have reported the rank of the lowest disease predicted by each method when the disease are sorted by their node degree in the network. We note that the number of unique diseases, obtained by the classical as well as the CAR-based indies in the top 50% of successfully predicted links, are generally those disease having the highest node degree in the network. However, the PRA index is capable of predicting some low-ranked diseases in the network.

## Supplementary information

Supplementary material 1 (pdf 498 KB)

## Data Availability

This study utilises data from UK Biobank project 31224—*Explanatory epidemiological models from genotype to phenotype*. Data from the UK Biobank can be accessed through an application process described in https://www.ukbiobank.ac.uk/enable-your-research.

## Code availability

The code, implemented in MATLAB, is available at our github repository https://github.com/azizfurqan/LPBN.
