# Supplementary material for: Multimorbidity prediction using link prediction
Source: Sci Rep. 2021 Aug 12;11:16392. doi: 10.1038/s41598-021-95802-0 (PMC8360941; doi:10.1038/s41598-021-95802-0)
Supplement: Supplementary file 1 — Supplementary material 1 (pdf 498 KB) [file 41598_2021_95802_MOESM1_ESM.pdf]

Supplementary Material:
Multimorbidity prediction using link prediction

Baseline Algorithms

**Index**	Unipartite Graph	Bipartite graph
CN	$Z_{uv}^{CN} = \lvert\{\Gamma(x) \cap \Gamma(y)\}\rvert$	$S_{uv}^{CN} = \left\lvert\{\widehat{\Gamma}(x) \cap \Gamma(y)\} \cup \{\Gamma(x) \cap \widehat{\Gamma}(y)\}\right\rvert$
JC	$Z_{uv}^{JC} = \frac{Z_{uv}^{CN}}{\lvert\Gamma(x) \cup \Gamma(y)\rvert}$	$S_{uv}^{JC} = \frac{S_{uv}^{CN}}{\lvert\Gamma(x) \cup \Gamma(y)\rvert}$
AA	$Z_{uv}^{AA} = \sum_{z \in \{\{\widehat{\Gamma}(x) \cap \Gamma(y)\} \cup \{\Gamma(x) \cap \widehat{\Gamma}(y)\}\}} \frac{1}{\log_2 \lvert\Gamma(z)\rvert}$	$S_{uv}^{AA} = \sum_{z \in \{\{\widehat{\Gamma}(x) \cap \Gamma(y)\} \cup \{\Gamma(x) \cap \widehat{\Gamma}(y)\}\}} \frac{1}{\log_2 \lvert\Gamma(z)\rvert}$
RA	$Z_{uv}^{RA} = \sum_{z \in \{\{\widehat{\Gamma}(x) \cap \Gamma(y)\} \cup \{\Gamma(x) \cap \widehat{\Gamma}(y)\}\}} \frac{1}{\lvert\Gamma(z)\rvert}$	$S_{uv}^{RA} = \sum_{z \in \{\{\widehat{\Gamma}(x) \cap \Gamma(y)\} \cup \{\Gamma(x) \cap \widehat{\Gamma}(y)\}\}} \frac{1}{\lvert\Gamma(z)\rvert}$
PA	$Z_{uv}^{PA} = \lvert\Gamma(x)\rvert \times \lvert\Gamma(y)\rvert$	$S_{uv}^{PA} = \lvert\Gamma(x)\rvert \times \lvert\Gamma(y)\rvert$
LCL	$Z_{uv}^{LCL} = \lvert\{(u,v) : (u,v) \in E, u \in \Gamma(y), v \in \Gamma(x)\}\rvert$	$S_{uv}^{LCL} = \lvert\{(u,v) : (u,v) \in E, u \in \Gamma(y), v \in \Gamma(x)\}\rvert$
CAR	$Z_{uv}^{CAR} = Z_{uv}^{CN} \times Z_{uv}^{LCL}$	$S_{uv}^{CAR} = S_{uv}^{CN} \times S_{uv}^{LCL}$
CJC	$Z_{uv}^{CJC} = \frac{Z_{uv}^{CAR}}{\lvert\Gamma(x) \cup \Gamma(y)\rvert}$	$S_{uv}^{CJC} = \frac{S_{uv}^{CAR}}{\lvert\Gamma(x) \cup \Gamma(y)\rvert}$
CAA	$Z_{uv}^{CAA} = \sum_{z \in \{\{\widehat{\Gamma}(x) \cap \Gamma(y)\} \cup \{\Gamma(x) \cap \widehat{\Gamma}(y)\}\}} \frac{\lvert\gamma(z)\rvert}{\log_2 \lvert\Gamma(z)\rvert}$	$S_{uv}^{CAA} = \sum_{z \in \{\{\widehat{\Gamma}(x) \cap \Gamma(y)\} \cup \{\Gamma(x) \cap \widehat{\Gamma}(y)\}\}} \frac{\lvert\gamma(z)\rvert}{\log_2 \lvert\Gamma(z)\rvert}$
CRA	$Z_{uv}^{CRA} = \sum_{z \in \{\{\widehat{\Gamma}(x) \cap \Gamma(y)\} \cup \{\Gamma(x) \cap \widehat{\Gamma}(y)\}\}} \frac{\lvert\gamma(z)\rvert}{\lvert\Gamma(z)\rvert}$	$S_{uv}^{CRA} = \sum_{z \in \{\{\widehat{\Gamma}(x) \cap \Gamma(y)\} \cup \{\Gamma(x) \cap \widehat{\Gamma}(y)\}\}} \frac{\lvert\gamma(z)\rvert}{\lvert\Gamma(z)\rvert}$
CPA	$Z_{uv}^{CPA} = \lvert\Gamma(x)\rvert \times \lvert\Gamma(y)\rvert$	$S_{uv}^{CPA} = \lvert\Gamma(x)\rvert \times \lvert\Gamma(y)\rvert$

Table S1: A list of baseline algorithms for unipartite and bipartite graphs. Here $\gamma(z)$ is the local community degree of z and corresponds to LCL.

Example of data split

In the following figure, we have explained how the observed links in a bipartite networks are partitioned into train and probe sets.

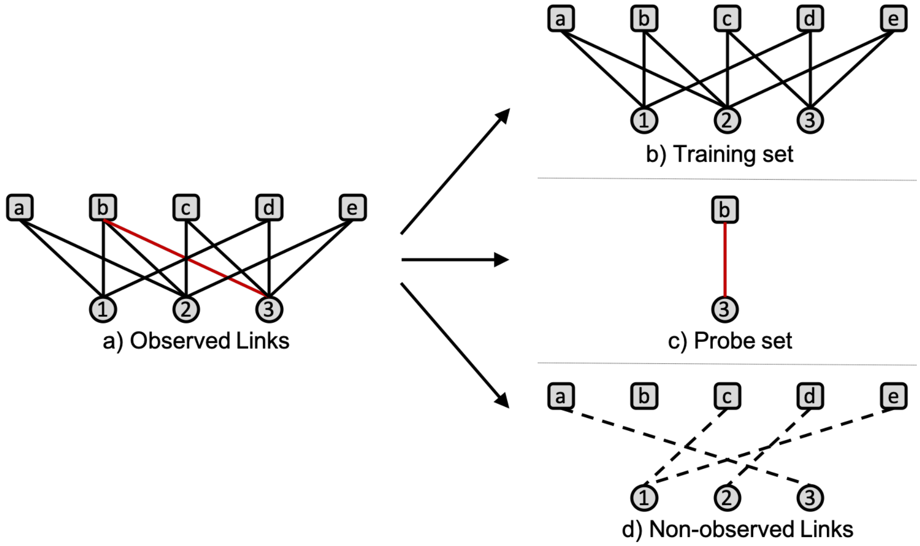

Figure S1: A simple example of the splitting the observed links E into training set E^T and probe set E^P is performed. Note that, for the case of bipartite network, the set of non-observed links is different. This is due to the fact that not all possible pairs of nodes in a bipartite network can interact with each other.

A simple Example

We demonstrate the process of computing similarity score using proposed method with the help of a numerical example on a small bipartite graph. Consider the bipartite patient-disease graph shown in Figure S2. Suppose the set {a,b,c,d,e} represents patients and the set {A,D,H,B,X} represent diseases. The text-box in the right of the figure shows the order in which a patient has developed the disease. Based on the data given in Figure S2, a conditional probability matrix

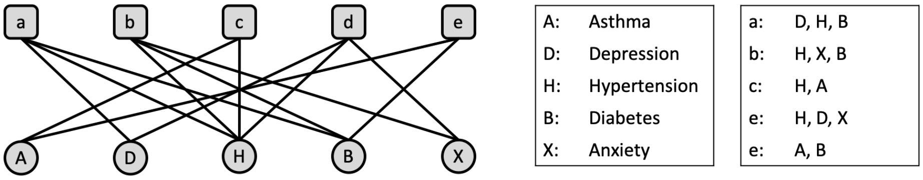

Figure S2: A simple bipartite graph of five patients and five diseases. A link between a patient and a disease represents that the patient has been diagnosed with that disease.

for diseases can be computed as follows:

$$\begin{array}{c c} & \begin{array}{ccccc} A & D & H & B & X \end{array} \\ \begin{array}{c} A \\ D \\ H \\ B \\ X \end{array} & \left[\begin{array}{ccccc} 0 & 0 & 0 & 0.50 & 0 \\ 0 & 0 & 0.50 & 0.50 & 0.50 \\ 0.25 & 0.25 & 0 & 0.50 & 0.50 \\ 0 & 0 & 0 & 0 & 0 \\ 0 & 0 & 0 & 0.50 & 0 \end{array}\right] \end{array}$$

Suppose we want to estimate the likelihood of the patient c developing the disease B. Since c already suffers from diseases A and H, and patients a, b, and e suffer from disease B, therefore there are three unique paths in the graph, (c, H, a, B), (c, H, b, B), and (c, A, e, B), that contribute to the similarity score, S_{cB}^{TPRA}. Therefore, the similarity score can be computed as:

$$\begin{aligned} S_{cB}^{TPRA} &= \sum_{(p',d') \in \{(a,H),(b,H),(e,A)\}} \frac{Pr(p' = B|p' = d')}{|\Gamma(p')|\,|\Gamma(d')|} \\ &= \frac{\Pr(a = b|a = H)}{|\Gamma(a)| \times |\Gamma(H)|} + \frac{\Pr(b = B|b = H)}{|\Gamma(b)| \times |\Gamma(H)|} + \frac{\Pr(e = B|e = A)}{|\Gamma(e)| \times |\Gamma(A)|} \\ &= \frac{0.5}{3 \times 4} + \frac{0.5}{3 \times 4} + \frac{0.5}{2 \times 2} \\ &= 0.2083 \end{aligned} \tag{1}$$

Results

Method	Precision	Recall	F-Score
CN	0.0064 ± 0.0000	1.0000 ± 0.0000	0.0127 ± 0.0000
JC	0.0350 ± 0.0117	0.3333 ± 0.1171	0.0633 ± 0.0211
AA	0.0470 ± 0.0174	0.4333 ± 0.1693	0.0845 ± 0.0311
RA	0.0476 ± 0.0185	0.4333 ± 0.1693	0.0856 ± 0.0332
PA	0.0196 ± 0.0189	0.1556 ± 0.1589	0.0347 ± 0.0338
LCL	0.0064 ± 0.0000	1.0000 ± 0.0000	0.0127 ± 0.0000
CAR	0.0085 ± 0.0065	**0.9444 ± 0.1757**	0.0166 ± 0.0121
CJC	0.0533 ± 0.0195	0.4000 ± 0.1304	0.0940 ± 0.0338
CAA	0.0471 ± 0.0174	0.4333 ± 0.1693	0.0846 ± 0.0311
CRA	0.0459 ± 0.0167	0.4333 ± 0.1693	0.0828 ± 0.0302
CPA	**0.0620 ± 0.0285**	0.4333 ± 0.1693	**0.1083 ± 0.0487**
PRA	0.0465 ± 0.0186	0.4333 ± 0.1693	0.0839 ± 0.0334

Table S2: Precision, Recall and F-Score for the NR dataset. The values are the average of 100 independent runs of the algorithm.

Method	Precision	Recall	F-Score
CN	0.0984 ± 0.0145	0.4655 ± 0.0481	0.1623 ± 0.0221
JC	0.0231 ± 0.0025	0.4014 ± 0.0291	0.0437 ± 0.0047
AA	0.1426 ± 0.0147	0.4210 ± 0.0419	0.2130 ± 0.0216
RA	0.1363 ± 0.0096	0.3966 ± 0.0278	0.2029 ± 0.0142
PA	0.0465 ± 0.0063	0.1358 ± 0.0184	0.0693 ± 0.0094
LCL	0.2353 ± 0.0060	0.6939 ± 0.0211	0.3515 ± 0.0091
CAR	0.2013 ± 0.0125	0.5973 ± 0.0394	0.3010 ± 0.0184
CJC	0.2311 ± 0.0081	0.6710 ± 0.0228	0.3437 ± 0.0119
CAA	0.2422 ± 0.0076	0.7014 ± 0.0218	0.3601 ± 0.0113
CRA	0.2487 ± 0.0095	0.7203 ± 0.0271	0.3698 ± 0.0141
CPA	0.2041 ± 0.0132	0.5966 ± 0.0389	0.3041 ± 0.0195
PRA	**0.2716 ± 0.0100**	**0.7885 ± 0.0263**	**0.4041 ± 0.0145**

Table S3: Precision, Recall and F-Score for the IC dataset. The values are the average of 100 independent runs of the algorithm.

**Method**	**Precision**	**Recall**	**F-Score**
CN	0.0553 ± 0.0031	**0.6297 ± 0.0478**	0.1016 ± 0.0053
JC	0.0383 ± 0.0116	0.1578 ± 0.0507	0.0616 ± 0.0188
AA	0.1584 ± 0.0126	0.5250 ± 0.0411	0.2433 ± 0.0193
RA	0.1586 ± 0.0133	0.5266 ± 0.0442	0.2438 ± 0.0204
PA	0.0514 ± 0.0143	0.1750 ± 0.0459	0.0795 ± 0.0218
LCL	0.0951 ± 0.0100	0.5625 ± 0.0448	0.1626 ± 0.0163
CAR	0.1492 ± 0.0106	0.5000 ± 0.0329	0.2299 ± 0.0161
CJC	0.1550 ± 0.0113	0.5203 ± 0.0369	0.2388 ± 0.0172
CAA	0.1592 ± 0.0132	0.5297 ± 0.0426	0.2448 ± 0.0201
CRA	0.1658 ± 0.0147	0.5500 ± 0.0476	0.2547 ± 0.0224
CPA	0.1506 ± 0.0102	0.5000 ± 0.0329	0.2315 ± 0.0155
PRA	**0.1828 ± 0.0164**	0.6078 ± 0.0553	**0.2810 ± 0.0253**

Table S4: Precision, Recall and F-Score for the GPCR dataset. The values are the average of 100 independent runs of the algorithm.

**Method**	**Precision**	**Recall**	**F-Score**
CN	0.0439 ± 0.0027	0.7348 ± 0.0290	0.0828 ± 0.0050
JC	0.0506 ± 0.0029	0.5174 ± 0.0239	0.0922 ± 0.0052
AA	0.0732 ± 0.0027	0.7420 ± 0.0275	0.1333 ± 0.0049
RA	0.0761 ± 0.0025	0.7737 ± 0.0286	0.1385 ± 0.0047
PA	0.0295 ± 0.0030	0.3007 ± 0.0300	0.0538 ± 0.0055
LCL	0.0665 ± 0.0033	0.7017 ± 0.0319	0.1214 ± 0.0059
CAR	0.0658 ± 0.0043	0.6928 ± 0.0363	0.1202 ± 0.0076
CJC	0.0688 ± 0.0030	0.6966 ± 0.0295	0.1252 ± 0.0054
CAA	0.0739 ± 0.0024	0.7468 ± 0.0255	0.1345 ± 0.0044
CRA	0.0765 ± 0.0029	0.7741 ± 0.0271	0.1392 ± 0.0053
CPA	0.0683 ± 0.0035	0.6898 ± 0.0352	0.1244 ± 0.0063
PRA	**0.0773 ± 0.0033**	**0.7829 ± 0.0325**	**0.1407 ± 0.0060**

Table S5: Precision, Recall and F-Score for the EZ dataset. The values are the average of 100 independent runs of the algorithm.

Method	Precision	Recall	F-Score
**CN**	0.0848 ± 0.0013	0.1123 ± 0.0017	0.0966 ± 0.0014
**LCL**	0.0783 ± 0.0010	0.1037 ± 0.0013	0.0893 ± 0.0011
**RA**	0.0876 ± 0.0011	0.1159 ± 0.0015	0.0997 ± 0.0013
**AA**	0.0868 ± 0.0013	0.1149 ± 0.0017	0.0989 ± 0.0014
**CRA**	0.0798 ± 0.0012	0.1056 ± 0.0015	0.0909 ± 0.0013
**CAA**	0.0794 ± 0.0011	0.1051 ± 0.0015	0.0905 ± 0.0013
**CPA**	0.0814 ± 0.0011	0.1078 ± 0.0014	0.0928 ± 0.0012
**JC**	0.0353 ± 0.0013	0.0468 ± 0.0017	0.0402 ± 0.0015
**PA**	0.0760 ± 0.0012	0.1025 ± 0.0018	0.0873 ± 0.0015
**CAR**	0.0814 ± 0.0011	0.1078 ± 0.0014	0.0928 ± 0.0012
**CJC**	0.0636 ± 0.0011	0.0842 ± 0.0015	0.0725 ± 0.0013
**PRA**	0.0929 ± 0.0013	0.1230 ± 0.0017	0.1058 ± 0.0015
**PROP**	**0.1052 ± 0.0014**	**0.1393 ± 0.0018**	**0.1199 ± 0.0016**

Table S6: Precision, Recall and F-Score for the MM1 dataset. The values are the average of 100 independent runs of the algorithm.

Method	Precision	Recall	F-Score
**CN**	0.0451 ± 0.0019	0.0498 ± 0.0021	0.0474 ± 0.0020
**LCL**	0.0430 ± 0.0018	0.0475 ± 0.0020	0.0452 ± 0.0019
**RA**	0.0445 ± 0.0019	0.0492 ± 0.0021	0.0467 ± 0.0020
**AA**	0.0446 ± 0.0019	0.0493 ± 0.0021	0.0468 ± 0.0020
**CRA**	0.0434 ± 0.0018	0.0480 ± 0.0020	0.0456 ± 0.0019
**CAA**	0.0433 ± 0.0018	0.0478 ± 0.0020	0.0454 ± 0.0019
**CPA**	0.0435 ± 0.0018	0.0480 ± 0.0020	0.0457 ± 0.0019
**JC**	0.0174 ± 0.0015	0.0192 ± 0.0017	0.0183 ± 0.0016
**PA**	0.0358 ± 0.0015	0.0467 ± 0.0020	0.0406 ± 0.0017
**CAR**	0.0435 ± 0.0018	0.0480 ± 0.0020	0.0457 ± 0.0019
**CJC**	0.0428 ± 0.0019	0.0473 ± 0.0020	0.0449 ± 0.0019
**PRA**	0.0443 ± 0.0019	0.0489 ± 0.0021	0.0465 ± 0.0019
**PROP**	**0.0621 ± 0.0020**	**0.0686 ± 0.0022**	**0.0652 ± 0.0021**

Table S7: Precision, Recall and F-Score for the MM2 dataset. The values are the average of 100 independent runs of the algorithm.

Method	Precision	Recall	F-Score
**CN**	0.1346 ± 0.0023	0.0749 ± 0.0013	0.0963 ± 0.0016
**LCL**	0.1020 ± 0.0020	0.0567 ± 0.0011	0.0729 ± 0.0014
**RA**	0.1344 ± 0.0023	0.0747 ± 0.0013	0.0960 ± 0.0016
**AA**	0.1343 ± 0.0023	0.0747 ± 0.0013	0.0960 ± 0.0017
**CRA**	0.1063 ± 0.0019	0.0590 ± 0.0011	0.0759 ± 0.0014
**CAA**	0.1044 ± 0.0018	0.0587 ± 0.0011	0.0751 ± 0.0014
**CPA**	0.1208 ± 0.0021	0.0671 ± 0.0012	0.0863 ± 0.0015
**JC**	0.0409 ± 0.0015	0.0227 ± 0.0009	0.0292 ± 0.0011
**PA**	0.0744 ± 0.0013	0.0479 ± 0.0009	0.0583 ± 0.0011
**CAR**	0.1208 ± 0.0021	0.0671 ± 0.0012	0.0863 ± 0.0015
**CJC**	0.0734 ± 0.0018	0.0411 ± 0.0013	0.0527 ± 0.0015
**PRA**	0.1423 ± 0.0020	0.0790 ± 0.0011	0.1016 ± 0.0014
**PROP**	**0.1655 ± 0.0028**	**0.0919 ± 0.0015**	**0.1182 ± 0.0020**

Table S8: Precision, Recall and F-Score for the MM3 dataset. The values are the average of 100 independent runs of the algorithm.

Method	MM1				MM2				MM3			
	TP	TN	FP	FN	TP	TN	FP	FN	TP	TN	FP	FN
**CN**	1942	28431	110385	15650	284	8678	6324	5686	1304	12217	8205	15829
**LCL**	1814	28077	110739	15778	284	8689	6313	5686	962	11865	8557	16171
**RA**	2007	28343	110473	15585	280	8685	6317	5690	1302	12213	8209	15831
**AA**	1976	28360	110456	15616	280	8683	6319	5690	1303	12189	8233	15830
**CRA**	1838	28269	110547	15754	283	8690	6312	5687	1009	11917	8505	16124
**CAA**	1830	28219	110597	15762	279	8688	6314	5691	997	11796	8626	16136
**CPA**	1860	28399	110417	15732	273	8682	6320	5697	1167	12080	8342	15966
**JC**	849	25618	113198	16743	117	8515	6487	5853	398	11310	9112	16735
**PA**	1773	22021	116795	15819	278	7501	7501	5692	829	10211	10211	16304
**CAR**	1860	28401	110415	15732	273	8682	6320	5697	1167	12080	8342	15966
**CJC**	1471	27846	110970	16121	273	8682	6320	5697	730	11510	8912	16403
**PRA**	2139	28257	110559	15453	284	8692	6310	5686	1366	12280	8142	15767
**PROP**	2387	29081	109735	15205	405	8814	6188	5565	1577	12491	7931	15556

Table S9: True Positive, False Positive, False Negative, and True Negative values for the three multimorbidity datasets.

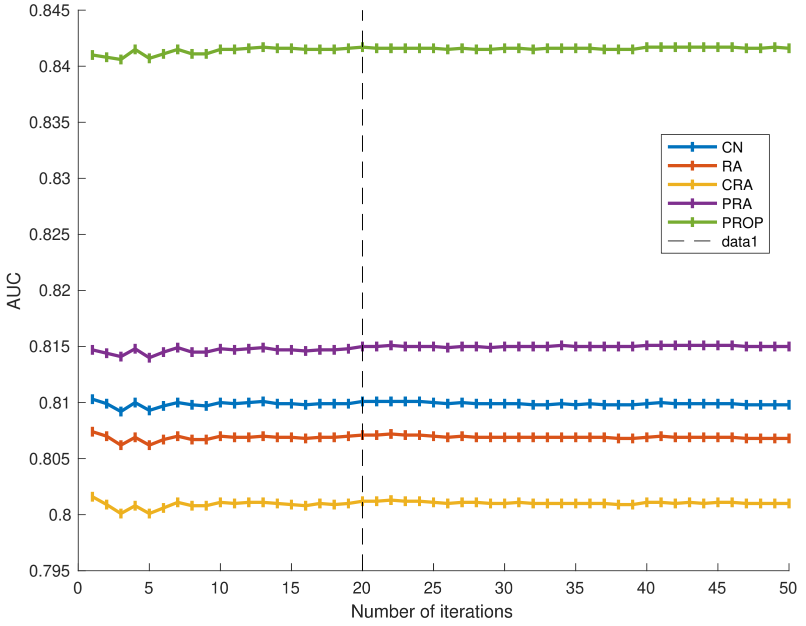

Figure S3: The average performance of five different methods on the MM2 dataset, measured by AUC for different number of runs. We can observe that there is very low variation in the AUC values and the results are stable after around 20 iterations. For visualization purposed, we have only included the results of the two proposed and three state-of-the art algorithms. We have observed that all the remaining indices follow a similar trend. These results are for the smallest size multimorbidity dataset. The performances of the prposed and alternate methods on the remaining datasets also converge around 20 iterations.

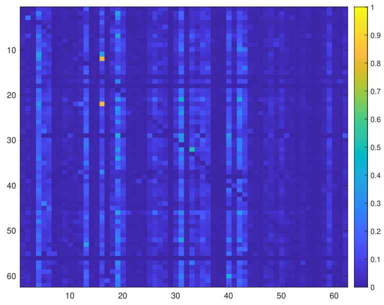

(a) MM2

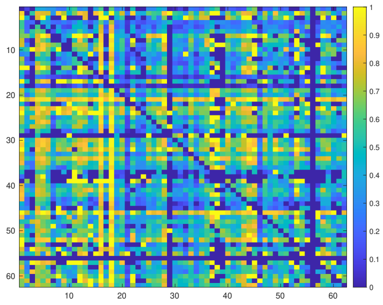

(b) MM2

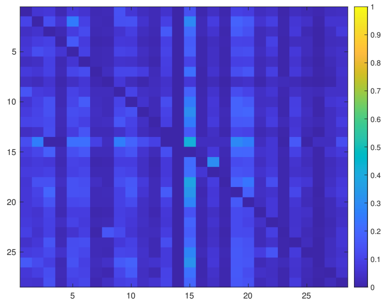

(c) MM3

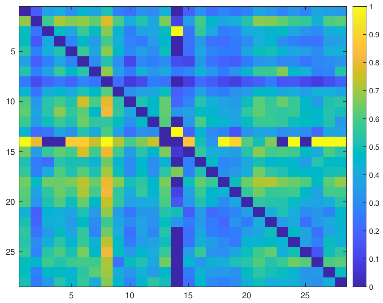

(d) MM3

Figure S4: Each matrix entry, $M(i,j)$, represents the conditional probability $Pr(patient = j|patient = i)$, ie., the probability that a patient is diagnosed with a disease j, given that he was already suffering from disease i. Left columns plot data for all patients, while in right columns, only those patients are considered who were diagnosed with both the diseases i and j. In other words, given that a patient has been diagnosed with both diseases i and j, how likely is that the patient was diagnosed with disease i before the patient was diagnosed with disease j. Warmer colors represent a higher probability.

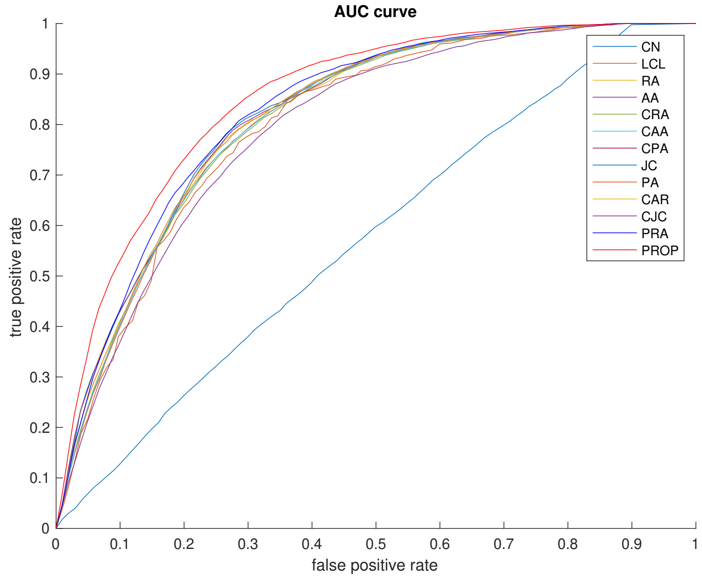

(a) MM2

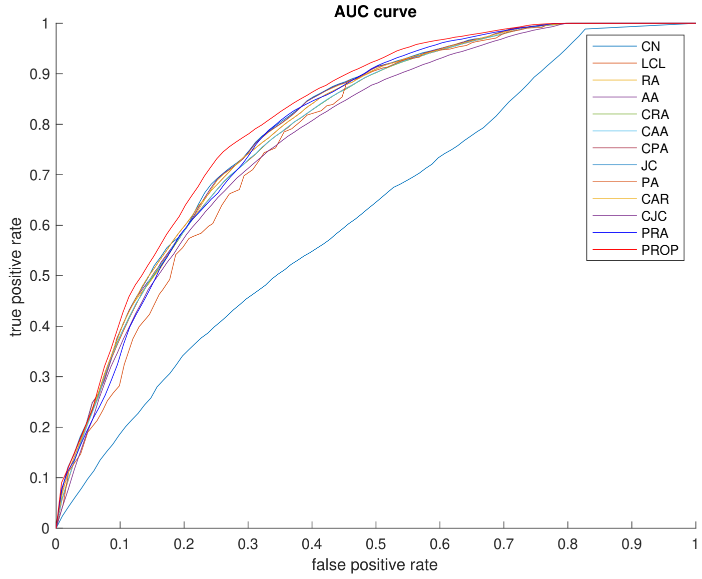

(b) MM3

Figure S5: Receiver operating characteristic curve for the performance of link prediction in datasets MM2 and MM3.

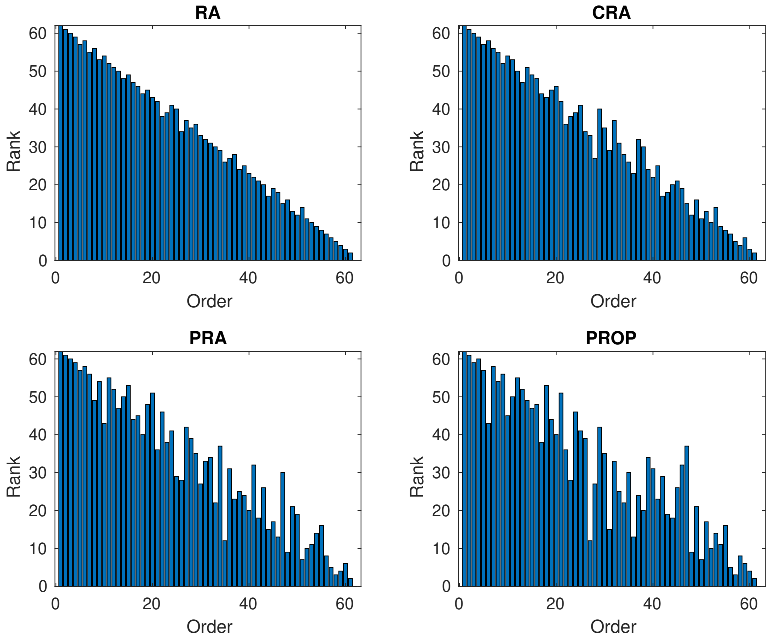

Figure S6: Prediction of new diseases for the multimorbidity dataset MM2. The x-axis shows the time when a new disease is predicted for the first time, while the y-axis shows the rank of the predicted disease when sorted using degree of disease in the network.

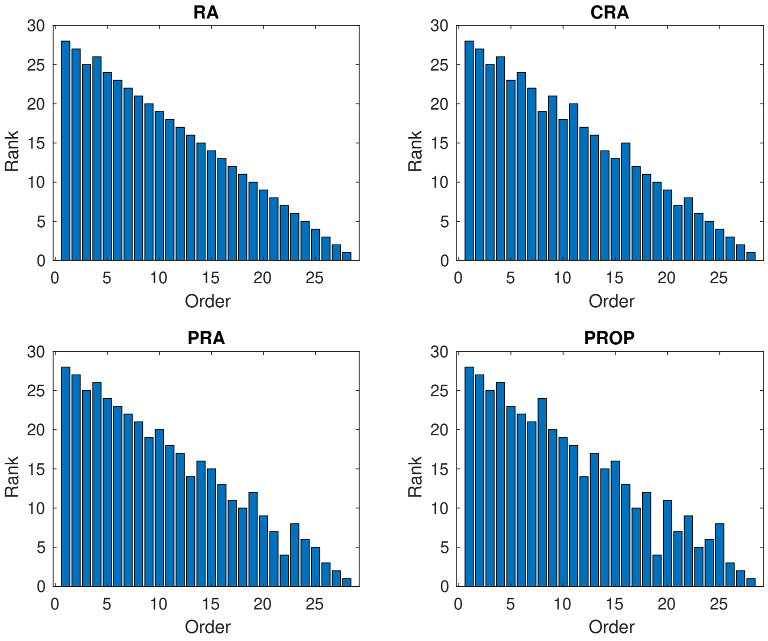

Figure S7: Prediction of new diseases for the multimorbidity dataset MM3. The x-axis shows the time when a new disease is predicted for the first time, while the y-axis shows the rank of the predicted disease when sorted using degree of disease in the network.

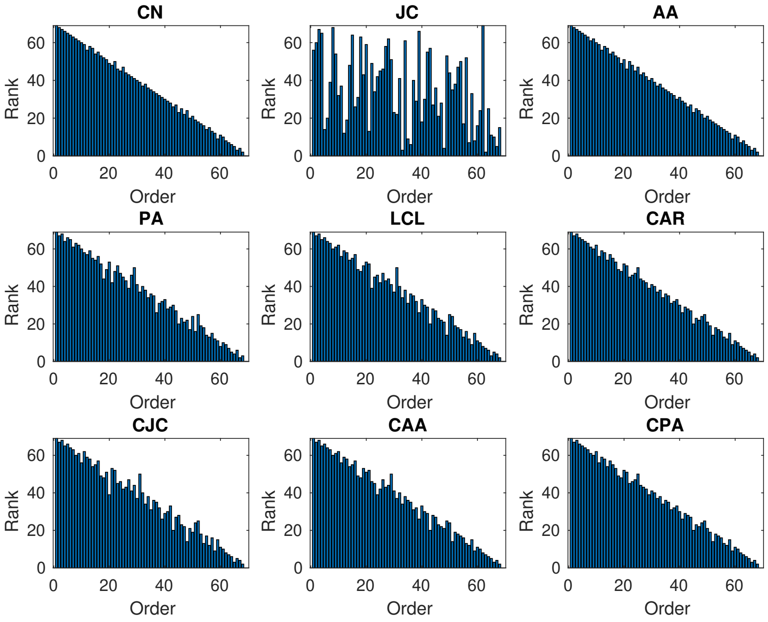

Figure S8: Prediction of new diseases for the multimorbidity dataset MM1. The x-axis shows the time when a new disease is predicted for the first time, while the y-axis shows the rank of the predicted disease when sorted using degree of disease in the network.

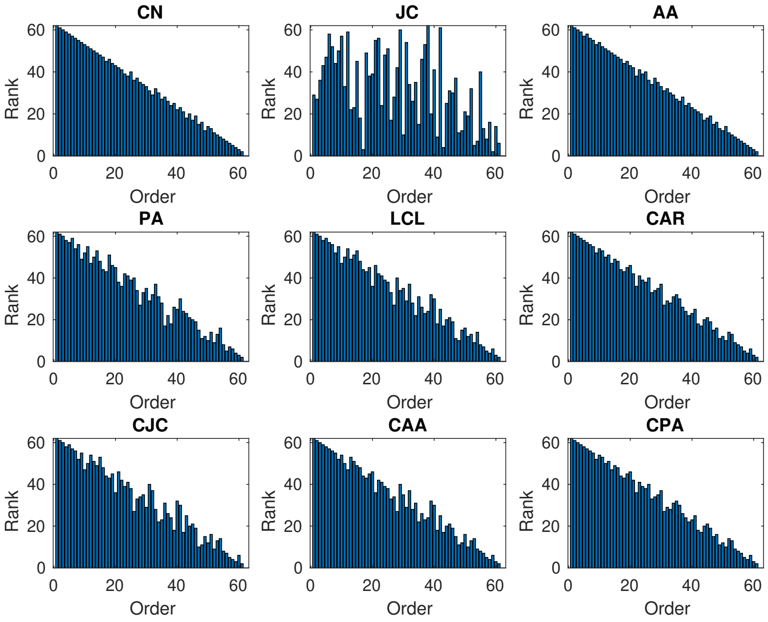

Figure S9: Prediction of new diseases for the multimorbidity dataset MM2. The x-axis shows the time when a new disease is predicted for the first time, while the y-axis shows the rank of the predicted disease when sorted using degree of disease in the network.

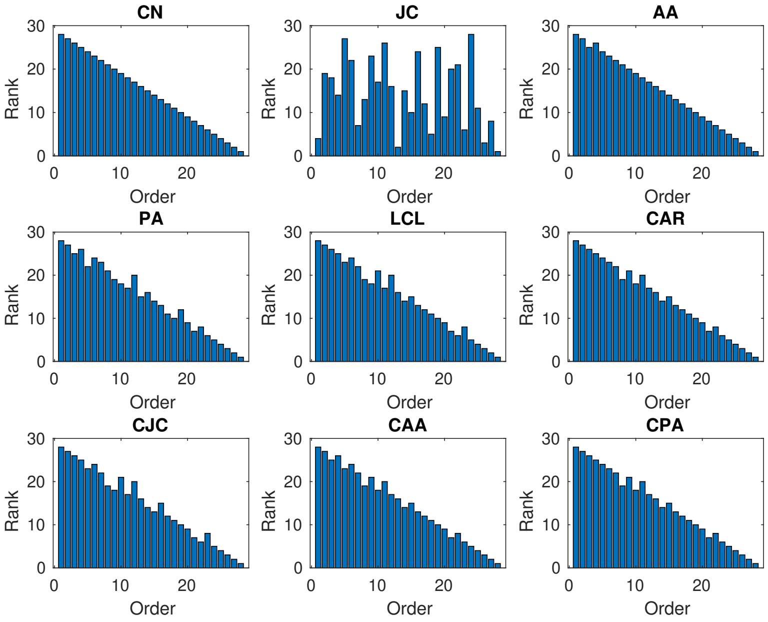

Figure S10: Prediction of new diseases for the multimorbidity dataset MM3. The x-axis shows the time when a new disease is predicted for the first time, while the y-axis shows the rank of the predicted disease when sorted using degree of disease in the network.

Multimorbidity dataset 1
Anxiety disorders (4197), Aplastic anaemias (333), Asthma (9544), Atrial fibrillation (8643), Bronchiectasis (1425), COPD (7200), Chronic sinusitis (798), Crohn's disease (591), Dementia (981), Depression (6432), Dermatitis (atopc/contact/other/unspecified) (1141), Diabetes (11022), Epilepsy (1707), Female pelvic inflammatory disease (1006), Glaucoma (1673), Heart failure (5289), Hyperparathyroidism (465), Hyperplasia of prostate (4546), Hypertension (26167), Hypo or hyperthyroidism (5854), Iron deficiency anaemia (4370), Irritable bowel syndrome (2315), Ischaemic stroke (2274), Liver fibrosis, sclerosis and cirrhosis (752), Meningitis (84), Migraine (1156), Multiple sclerosis (328), Myocardial infarction (8066), Osteoporosis (3632), Parkinson's disease (643), Primary malignancy Bladder (1146), Primary malignancy bone and articular cartilage (84), Primary malignancy brain, other CNS and Intracranial (229), Primary malignancy breast (2885), Primary malignancy cervical (79), Primary malignancy kidney and ureter (712), Primary malignancy liver (216), Primary malignancy lung and trachea (1881), Primary malignancy malignant melanoma (628), Primary malignancy mesothelioma (133), Primary malignancy multiple independent sites (176), Primary malignancy oesophageal (532), Primary malignancy Oropharyngeal (434), Primary malignancy other organs (2230), Primary malignancy other skin and subcutaneous tissue (3016), Primary malignancy ovarian (560), Primary malignancy pancreatic (438), Primary malignancy prostate (2190), Primary malignancy stomach (454), Primary malignancy testicular (51), Primary malignancy thyroid (145), Primary malignancy uterine (482), Primary malignancy biliary tract (239), Primary malignancy colorectal and anus (2230), Rheumatoid arthritis (2257), Schizophrenia, schizotypal and delusional disorders (473), Secondary malignancy adrenal gland (379), Secondary malignancy bone (2279), Secondary malignancy bowel (262), Secondary malignancy brain. Other CNS and intracranial (927), Secondary malignancy lung (2124), Secondary malignancy lymph nodes (4026), Secondary malignancy other organs (1681), Secondary malignancy pleura (688), Secondary malignancy retroperitoneum and peritoneum (1367), Stable angina (10426), Stroke NOS (1392), Tuberculosis (133), Unstable angina (3825).

Table S10: List of unique diseases in multi-morbidity dataset (MM1) along with count of number of patients that are diagnosed with that disease. There are a total of 69 unique diseases in this network.

Multimorbidity dataset 2
Acuterenalfailure (227), Alcoholdependence (713), Alcoholliverdisease (76), Asthmalonglist (2983), Atrialfibrillation (1222), Atriskoffalls (1367), Bipolar (158), Bladdercancer (134), Benign Prostatic Hyperplasia (562), Breastcancer (460), Bronchiectasis (214), Chronicbronchitis (367), Ckd (2519), Coeliac (151), Colorectalcancer (245), Chronic obstructive pulmonary disease (1463), Crohn (142), Dementia (571), Depresion (5114), Diverticulardisease (1813), Duodenalulcer (577), Emphysem (90), Epilepsy (413), Gastriculcer (175), Glaucoma (965), Gord (2050), Gout (1083), Heartfailure (635), Human immunodeficiency virus (12), Hyperlipidaemia (1651), Hypertension (6035), Hyperthyroidismdirty (444), Hypothyroidismdirty (2057), Irritable bowel syndrome (1877), Ischaemic heart disease (2192), Irondef (1492), Lungcancer (56), Multiplemyelomadirty (27), Multiplesclerosis (80), Nontypediabetes (2796), Obesitydirty (753), Osteoarthritis (4157), Osteoporosis (1588), Pacemaker (277), Parkinsonsdiseasev (123), Pepticulcerdirty (193), Peripheralvasculardisease (522), Pneumoniadirty (820), Prostatecancerdirty (336), Psoriasis (1128), Pulmonaryembolismdirty (399), Renalcolicdirty (533), Renalcancerdirty (46), Rheumatoidarthritis (350), Schizophrenia (143), Sicklecelldisease (0), Systemic lupus erythematosus (68), Sleepapnoea (333), Strokeandtia (1519), Typediabetes (256), Ulcerativecolitis (164), Venous thromboembolism (780).

Table S11: List of unique diseases in multi-morbidity dataset (MM2) along with count of number of patients that are diagnosed with that disease. There are a total of 62 unique diseases in this network.

Multimorbidity dataset 3
Hypertension (28891), Ischaemic heart disease (17933), Diabetes (13037), Malignant neoplasms (13016), Asthma (10720), Atrial fibrillation (10487), Chronic bronchitis (8341), Depression (7508), Heart failure (6682), Chronic kidney disease (6553), Hypothyroidism (6352), Peripheral vascular disease (5388), Anxiety (4816), Stroke (transient ischaemic attack) (4729), Osteoporosis (4232), Duodenal and gastric ulcer (3790), Sleep apnoea (3035), Rheumatoid arthritis (2706), Osteoarthritis (2619), Epilepsy (1977), Bronchiectasis (1672), Inflammatory bowel disease (1571), Chronic liver disease (1564), Hyperthyroidism (1225), Dementia (1125), Parkinsons disease (781), Severe mental illness (549), Human immunodeficiency virus (31).

Table S12: List of unique diseases in multi-morbidity dataset (MM3) along with count of number of patients that are diagnosed with that disease. There are a total of 28 unique diseases in this network.

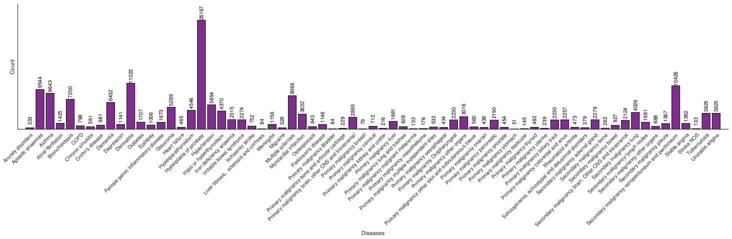

(a) MM1

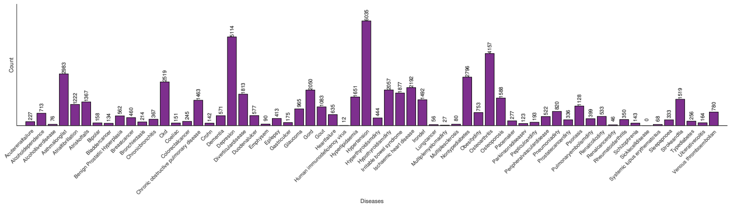

(b) MM2

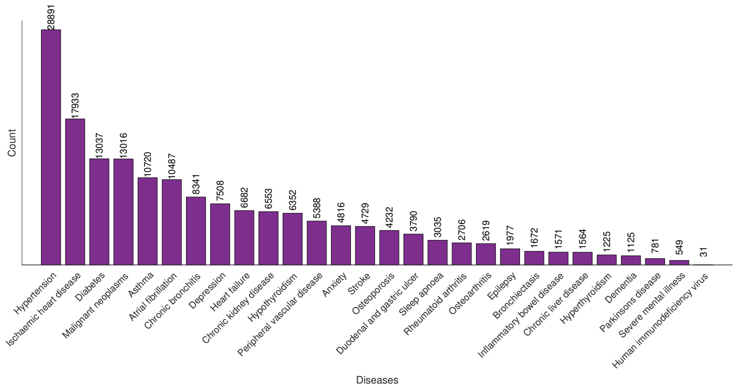

(c) MM3

Figure S11: Bar chart of number of patients for each unique diseases in all the multi-morbidity datasets.
